# Safety and Efficacy of DTX401, an AAV8‐Mediated Liver‐Directed Gene Therapy, in Adults With Glycogen Storage Disease Type I a (GSDIa)

**DOI:** 10.1002/jimd.70014

**Published:** 2025-03-10

**Authors:** David A. Weinstein, Terry G. Derks, David F. Rodriguez‐Buritica, Ayesha Ahmad, María‐Luz Couce, John J. Mitchell, Rebecca Riba‐Wolman, Malaya Mount, Julieta Bonvin Sallago, Katalin M. Ross, Melanie M. van der Klauw, Foekje de Boer, Caroline van der Schaaf, Heather Saavedra, Miguel Martínez‐Olmos, Elvis Atanga, Asad Hosseini, Deepali Mitragotri, Eric Crombez

**Affiliations:** ^1^ Department of Pediatrics University of Connecticut Farmington Connecticut USA; ^2^ Section of Metabolic Diseases Beatrix Children's Hospital, University Medical Center Groningen, University of Groningen Groningen the Netherlands; ^3^ Department of Pediatrics, Division of Medical Genetics McGovern Medical School at the University of Texas Health Science Center at Houston (UTHealth Houston) and Children's Memorial Hermann Hospital Houston Texas USA; ^4^ University of Michigan Ann Arbor Michigan USA; ^5^ University Clinical Hospital of Santiago de Compostela, IDIS, CIBERER Santiago de Compostela Spain; ^6^ Montreal Children's Hospital Montreal Quebec Canada; ^7^ Department of Endocrinology University of Groningen, University Medical Center Groningen Groningen the Netherlands; ^8^ Ultragenyx Pharmaceutical Inc. Novato California USA

**Keywords:** DTX401, gene therapy, glycogen storage disease, GSD, GSDIa

## Abstract

Glycogen storage disease type Ia (GSDIa) is a rare, life‐threatening, inherited carbohydrate metabolism disorder caused by glucose‐6‐phosphatase (G6Pase) deficiency, which is essential for glycogenolysis and gluconeogenesis. GSDIa management includes a strict medically prescribed diet that typically includes daily uncooked cornstarch doses, including overnight, to maintain euglycemia. DTX401 is an investigational adeno‐associated virus serotype 8 vector expressing the human *G6PC1* gene that encodes G6Pase. This open‐label, phase 1/2, dose‐escalation, 52‐week gene therapy trial evaluated the safety and efficacy of a single DTX401 infusion in 12 adults with GSDIa (ClinicalTrials.gov Identifier: NCT03517085). Three participants in Cohort 1 received DTX401 2.0 × 10^12^ genome copies (GC)/kg, and three participants each in Cohorts 2, 3, and 4 received 6.0 × 10^12^ GC/kg. Corticosteroids were administered to mitigate vector‑induced inflammatory response. All participants experienced a treatment‐emergent adverse event (TEAE) and a related TEAE. No participant experienced a dose‐limiting toxicity, TEAE leading to study discontinuation, TEAE leading to death, or serious treatment‐related TEAE. Mean (SD) time to hypoglycemia in minutes/gram of carbohydrate during a controlled fasting challenge was 5.0 (1.6) at baseline and 6.9 (2.7) at Week 52, a mean (SD) increase of 46% (72%). Mean total daily cornstarch intake was 284 g at baseline and 85 g at Week 52 in the 10 participants with available values at both time points, a mean (SD) total daily cornstarch intake reduction of 68% (20%); *p* < 0.001. DTX401 showed a favorable safety and efficacy profile at Week 52. Participants in all cohorts showed significant cornstarch need reductions from baseline to Week 52.

## 1 Introduction

Glycogen storage disease type Ia (GSDIa, OMIM #232200) is a life‐threatening, rare, inherited disorder of carbohydrate metabolism with a prevalence of approximately 1 in 100 000 [1]. GSDIa results from biallelic pathogenic variants in the *G6PC1* gene (OMIM *613742) that encodes glucose‐6‐phosphatase (G6Pase) in the liver, kidneys, and intestines [1, 2]. Patients with GSDIa experience severe fasting intolerance biochemically associated with hypoketotic hypoglycemia, lactic acidosis, hyperlipidemia, and hyperuricemia [3]. Acute GSDIa complications include severe hypoglycemia, lactic acidosis, nephrolithiasis, gout, pancreatitis, and bleeding diathesis [4]. Chronic GSDIa complications may include short stature, delayed puberty, hepatic adenomas that may evolve into carcinoma, kidney dysfunction with glomerular hyperfiltration that can progress to microalbuminuria and proteinuria due to focal segmental glomerular sclerosis, osteoporosis, and rarely, a small fiber peripheral neuropathy [2, 4, 5, 6].

Management of GSDIa includes a strict medically prescribed diet to provide a continuous exogenous source of glucose to (1) compensate for deficient endogenous glucose production, (2) maintain glucose homeostasis, (3) reduce or prevent acute/chronic complications, and (4) preserve quality of life as much as possible [3]. The prescribed diet typically includes regular, individualized uncooked cornstarch doses, including overnight [7]. Complication risk can be decreased but not fully prevented by the diet if exceptional metabolic control can be achieved [8]. Liver transplantation, currently the only alternative treatment option, is reserved for selected patients due to associated morbidities and mortality.

Despite treatment, a subset of patients with GSDIa remains at risk for ongoing metabolic instability. Patients have a persistent and permanent risk of severe hypoglycemia if cornstarch doses are missed, especially at night [3, 9]. Most patients consider their disease to be of moderate severity, but with difficult dietary treatment challenges; however, significant anxiety is chronically present due to fear of hypoglycemia [10]. Extending fasting time and reducing the risk of severe hypoglycemia are critical for improving the prognosis and quality of life in patients with GSDIa, since even with appropriate adherence to current management approaches, short‐ and long‐term complications almost universally occur [11].

Gene therapies have been evaluated in several GSDIa animal models including mice and the naturally occurring dog model [12, 13, 14, 15, 16, 17]. Following the demonstration of improved fasting tolerance, improved survival, and few complications in the preclinical studies, this first in human gene therapy trial for GSDIa was performed. In this manuscript, safety and efficacy results are reported from this trial in adults with genetically confirmed GSDIa.

## 2 Materials and Methods

### 2.1 Trial Design

**FIGURE 1:**
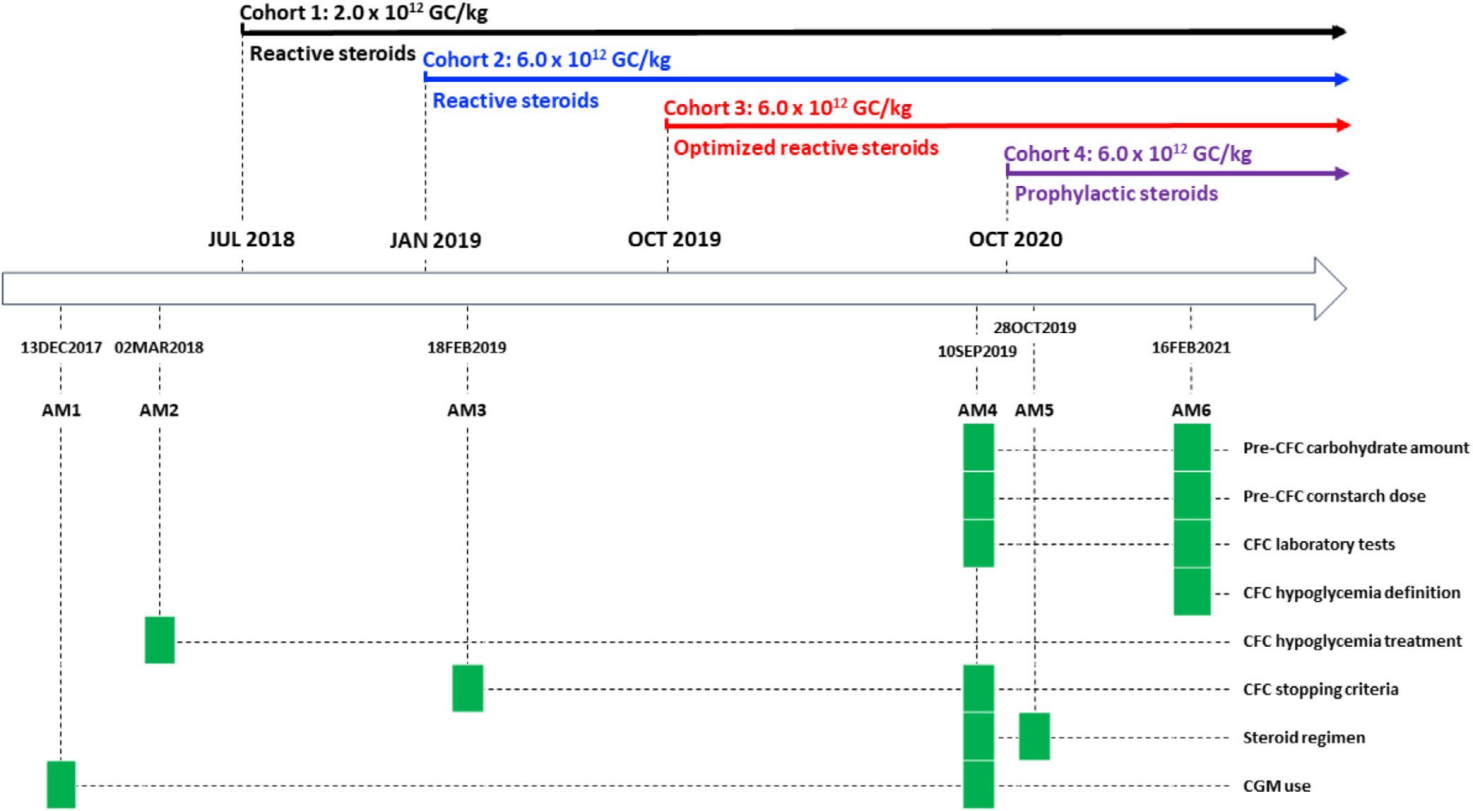
DTX401 phase 1/2 study schema and protocol amendments. AM, amendment. CFC, controlled fasting challenge. CGM, continuous glucose monitoring.

This was a phase 1/2, open‐label, dose‐escalation, interventional 52 week trial (NCT03517085) of DTX401 in adults with GSDIa, after which participants transitioned to a long‐term follow‐up study (NCT03970278). The study was conducted at seven centers (four in the United States and one each in Canada, Spain, and the Netherlands). The initial study design included three different single‐dose cohorts of DTX401 with three participants per cohort. Six protocol amendments were implemented throughout the study and were introduced to address modifications to the following topics: (a) the corticosteroid regimen, (b) the controlled fasting challenge (CFC) protocol, (c) continuous glucose monitoring (CGM), and (d) endpoints (Figure 1). DTX401 was administered through a peripheral intravenous infusion over 30 min. The dose was calculated by body weight on the day of the infusion up to a maximum of 100 kg.

### 2.2 Vector Description

DTX401 (pariglasgene brecaparvovec) is a codon‐optimized human wild‐type *G6PC1* transgene with expression driven by both the native *G6PC*‐specific promoter and enhancer elements encapsidated within a nonreplicating recombinant adeno‐associated virus serotype 8 (AAV8) vector. DTX401 was produced by triple plasmid DNA transfection of a human embryonic kidney 293 master cell bank plasmid pDTX.hG6PCco.401 (DTC161), the DTX401 expression plasmid containing the human *G6PC* gene, plasmid pAAV2‐8.KanR (p2123‐FH), the AAV helper plasmid containing the AAV2 replication and AAV8 capsid wild‐type genes, and plasmid pAdDeltaF6(Kan), a helper adenovirus plasmid.

### 2.3 Participants

Adults ≥ 18 years of age with molecularly confirmed GSDIa with biallelic pathogenic *G6PC1* variants and a history of at least one hypoglycemic event with glucose < 60 mg/dL (< 3.33 mmol/L) were eligible for the study. Exclusion criteria included hospitalization for severe hypoglycemia during the f4 weeks before screening, screening or baseline (Day 0) glucose < 60 mg/dL (< 3.33 mmol/L), alanine aminotransferase (ALT) or aspartate aminotransferase (AST) > the upper limit of normal (ULN), total bilirubin > 1.5 × ULN, or alkaline phosphatase > 2.5 × ULN, triglycerides ≥ 1000 mg/dL, creatinine > 2.0 mg/dL, an anti‐AAV8 neutralizing antibody titer ≥ 1:5, previous liver transplant, presence of liver adenoma > 5 cm in size, or presence of liver adenoma > 3 cm and ≤ 5 cm in size with a documented annual growth rate of ≥ 0.5 cm per year.

### 2.4 Trial Endpoints

The primary endpoint was to evaluate safety by the incidence of adverse events (AEs), including dose‐limiting toxicities (DLTs) at each dose level, treatment‐emergent AEs (TEAEs), and serious AEs (SAEs) for each cohort, assessed by severity and relationship to DTX401. DLTs were defined as any AE/SAE ≥ Grade 3 that was considered by the investigator and/or sponsor to be related to DTX401. Any abnormal laboratory test results (clinical chemistry [including liver function tests], hematology, coagulation panel, urinalysis, other laboratory parameters), including those that worsened from baseline or were felt to be clinically significant in the medical/scientific judgment of the investigator, were recorded as AEs or SAEs. Any clinically significant safety findings associated with GSDIa were not reported as AEs unless judged by the study investigator to be more severe than expected for the condition of the participant.

The secondary endpoint was change from baseline in time to first hypoglycemic event (defined as glucose < 54 mg/dL [< 3.0 mmol/L]) during the controlled fasting challenge (CFC) at 12, 24, and 52 weeks after DTX401 administration. The CFC required a 24‐h hospital stay, was performed at night, and assessed fasting tolerance after a standardized dinner and a small cornstarch dose to minimize the post‐prandial insulin response. Blood samples were obtained at established glucose concentration‐dependent intervals to measure analytes, including glucose, insulin, and lactate concentrations. Stopping criteria for the CFC were (a) glucose < 54 mg/dL (changed from < 60 mg/dL with a protocol amendment), (b) duration of 15 h, or (c) symptoms of hyperlactatemia or hypoglycemia at the discretion of the physician or participant.

During the study, it was observed that both prednisone administration and DTX401 can affect glucose homeostasis and the strict dietary and cornstarch regimens participants consumed; therefore, for participants enrolled in Cohorts 3 and 4, CGM was added to the trial protocol. CGM is increasingly becoming the standard of care for patients with GSDIa to minimize the risk of hypoglycemia as cornstarch therapy is weaned. [18] Using CGM, a hypoglycemic event was defined as a series of at least two sensor glucose values < 60 mg/dL (< 3.3 mmol/L), lasting at least 15 min, with no intervening values ≥ 60 mg/dL (≥ 3.3 mmol/L). The end of a hypoglycemic event was defined as a minimum of 15 consecutive minutes with ≥ 2 sensor glucose values ≥ 60 mg/dL (≥ 3.3 mmol/L) and at least 10 mg/dL (0.6 mmol/L) above the nadir of the event or at the end of a week.

Patient‐reported quality of life was assessed with the Patient Global Impression of Change (PGIC) and the Patient Global Impression of Severity (PGIS), two assessment tools designed to capture changes in quality of life over time and at specific timepoints. PGIC was assessed at Week 52 in Cohorts 2, 3, and 4, and PGIS was assessed at Baseline and at Weeks 6, 12, 24, and 52 in Cohorts 3 and 4.

Key changes in study protocol amendments are shown in Table 1.

**TABLE 1:** Key changes in study protocol amendments.

Protocol amendment	1	2	3	4	5	6
Date of amendment	13‐Dec‐2017	02‐Mar‐2018	18‐Feb‐2019	10‐Sep‐2019	28‐Oct‐2019	16‐Feb‐2021
Pre‐CFC carbohydrate amount Original protocol: 20–30 g	NC	NC	NC	Changed to 15–20 g	NC	Changed to a personalized meal with a target carbohydrate range and an overall composition in protein, fats, and dietary fiber as close as possible to the most current dinner prescription, but not higher than the carbohydrate content of the dinner consumed at the baseline fasting challenge for each participant
Pre‐CFC cornstarch dose Original protocol: 35 g	NC	NC	NC	Changed to 5 g	NC	Match most recent cornstarch prescription and timing after dinner
Pre‐CFC lab tests and critical samples Original protocol: Glucose, lactate, standard clinical chemistry, hematology, coagulation panel, urinalysis, AST, and ALT	NC	NC	NC	Lipid levels, cortisol, fatty acid, glucagon, insulin, and ketone levels added	NC	ACTH, C‐peptide, growth hormone, IGFBP1, and alanine added at beginning and end of CFC
CFC hypoglycemia definition Original protocol: < 60 mg/dL (< 3.33 mmol/L)	NC	NC	NC	NC	NC	< 54 mg/dL (< 3.0 mmol/L)
Prednisone regimen Original protocol: 40 mg/day after ALT elevation	NC	NC	NC	Reactive steroid starting dose changed from 40 to 60 mg/day. The criterion for initiating steroids was modified to an increase in ALT compared with baseline or recently drawn levels. Language to allow steroid regimen modification if ALT levels did not normalize during steroid taper added.	Cohort 4 added to assess prophylactic steroid regimen 60 mg/day beginning on Day 1	NC
CGM Original protocol: yes	CGM removed	NC	NC	CGM added for all currently enrolled participants	NC	NC
Changes not captured above	N/A	Instructions for treating hypoglycemia after CFC added	CFC stopping criteria changed to include symptoms of hypoglycemia added	CFC stopping criteria changed to remove symptoms of hypoglycemia. PGIS and PGIC health‐related quality of life assessments added	N/A	CFC assessment was removed at Week 6 and made optional at Week 12

Abbreviations: ACTH, adrenocorticotropic hormone; ALT, alanine aminotransferase; AST, aspartate aminotransferase; CFC, controlled fasting challenge; CGM, continuous glucose monitoring; IGFBP1, insulin‐like growth factor binding protein 1; N/A, not applicable; NC, no change from previous; PGIC, patient global impression of change; PGIS, patient global impression of severity.

### 2.5 Trial Oversight

The study was conducted in accordance with the International Council for Harmonisation of Technical Requirements for Pharmaceuticals for Human Use (ICH) Good Clinical Practice (GCP) guidelines and in accordance with the Declaration of Helsinki. The protocol was approved by the institutional review board at each participating center. All participants provided written informed consent before participation in the trial. An external data and safety committee monitored participant safety.

### 2.6 Statistical Analysis

The Full Analysis Set was used for all analyses and consisted of all enrolled participants who received DTX401. Descriptive analyses included summary statistics, graphs, and data listings. No inferential testing was planned. CGM analysis was performed as described elsewhere, with slight modifications [18, 19]. For clinical laboratory parameters with continuous results, values and changes from baseline were summarized by study visit. For clinical laboratory parameters with categorical results, data were included in a listing; these parameters were either non‐critical for analysis or were collected only at screening. The nominal *p*‐value corresponding to a 1‐sample t‐test to determine statistical significance of mean percent change from baseline to Week 52 in cornstarch was calculated.

## 3 Results

### 3.1 Characteristics of the Study Participants

A total of 26 participants were screened; 12 participants enrolled and were dosed, with three in each of the four treatment cohorts. Reasons participants failed screening were transaminase elevation (*n* = 6), triglycerides ≥ 1000 mg/dL (*n* = 1), hepatic adenomas > 5 cm in size (*n* = 2), and anti‐AAV8 neutralizing antibody titer ≥ 1:5 (*n* = 5). First enrollment was 18 May 2018, and the Week 52 visit for Participant 12 was 02 November 2021. Due to the COVID‐19 pandemic, participants 6 and 7 did not complete the end of study visits until Study Days 569 and 533, respectively; as a result, adverse event data were collected to these timepoints for these participants. All other participants completed the Week 52 end of study visits between Study Days 358 and 385.

Table 2 summarizes demographics and baseline characteristics. At baseline, nephrolithiasis, gastrostomy, and liver biopsy each were reported for five participants (42%), and hyperlipidemia, nephropathy, osteoporosis, hepatomegaly, and hepatic adenoma each were reported for four participants (33%). In nine participants, cornstarch was initiated the same year as diagnosis. In two participants, continuous nocturnal drip feeds were initially used but changed to cornstarch regimens as management strategies evolved from when these participants were initially diagnosed with GSDIa in the 1960s [20]. For one participant, the timing of cornstarch initiation could not be obtained. At baseline, prescribed overnight dietary regimens/support included using extended‐release waxy maize cornstarch (*n* = 5), traditional cornstarch (*n* = 6), and a continuous overnight gastric drip feeding (*n* = 1). Median sleep time before awakening to take cornstarch was 6.0 (range 2–9) hours.

**TABLE 2:** Baseline demographics and planned prednisone treatment regimen.

	Cohort 1 (2.0 × 10^12^ GC/kg) reactive steroids (6 weeks, at a starting dose of 40 mg/day, after ALT elevation)			Cohort 2 (6.0 × 10^12^ GC/kg) reactive steroids (6 weeks, at a starting dose of 40 mg/day, after ALT elevation)		
Participant number	1	2	3	4	5	6
Gender	Male	Female	Male	Male	Male	Male
Age (years)	28	57	51	31	19	39
*G6PC* genotype	p.Gln347*/p.Gln27Argfs*9	p.Gln347*/(homozygous)	p.Gln347*/p.Arg83Cys	p.Tyr128Thrfs*3/(homozygous)	p.Arg83Cys/p.Thr108lle	p.Trp63*/p.Gln27Argfs*9
Screening weight (kg)	57	59	80	114	74	93
Total GC	1.14E+14	1.19E+14	1.60E+14	6.00E+14	4.47E+14	5.58E+14
Baseline total daily cornstarch dose	405 g	171 g^a^	269 g	325 g	270 g	329 g
Cornstarch doses per day (night [12 AM to 6 AM] doses)	7 (1)	3 (1)	7 (2)	5 (1)	6 (1)	8 (2)
Baseline time to hypoglycemia (hours)	3.8	4.0	5.4	6.1	3.6	3.8

	Cohort 3 (6.0 × 10^12^ GC/kg) optimized reactive steroids (7 weeks, at a starting dose of 60 mg/day, after ALT elevation)			Cohort 4 (6.0 × 10^12^ GC/kg) prophylactic steroids (8 weeks, at a starting dose of 60 mg/day, starting on Day 1)		
Participant number	7	8	9	10	11	12
Gender	Female	Female	Male	Male	Female	Male
Age (years)	18	42	18	31	22	22
*G6PC* Genotype	p.Arg83Cys/(homozygous)	p.Arg83Cys/(homozygous)	p.Gln27Argfs*9/p.Arg83Cys	p.Arg170*/p.Phe327del	p.Arg83Cys/(homozygous)	p.Gln27Argfs*9/p.Lys263Argfs*38
Screening weight (kg)	63	119	87	84	65	56
Total GC	3.78E+14	6.00E+14	5.21E+14	5.03E+14	3.90E+14	3.37E+14
Baseline total daily cornstarch dose	373 g	240 g	324 g	240 g	260 g	335 g
Cornstarch doses per day (night [12 AM to 6 AM] doses)	5 (0)	6 (0)	7 (1)	6 (2)	6 (2)	7 (1)
Baseline time to hypoglycemia (hours)	1.4^b^	3.6^b^	1.9^b^	3.5^b^	1.7^b^	2.4^b^

Abbreviations: ALT, alanine aminotransferase; GC, genome copies.

^a^ Overnight continuous gastrostomy tube feed.

^b^ Fasting challenge performed with 5 g of cornstarch.

### 3.2 Safety Outcomes

There were no infusion‐associated reactions in any of the 12 participants during DTX401 administration. TEAEs pertaining to liver enzyme elevations were reported in all participants. The most commonly reported other adverse events (number of participants, %) were headache (8, 67%), diarrhea (5, 42%), and vomiting (5, 42%). While all participants experienced elevated triglycerides, there were seven episodes of hypertriglyceridemia reported as AEs in three participants (25%).

All participants experienced at least one TEAE considered by the investigator to be related to study drug; all treatment‐related TEAEs were mild or moderate in severity. No participant experienced a DLT, TEAE leading to study discontinuation, TEAE leading to death, or serious treatment‐related TEAE.

Five serious TEAEs were reported in four participants (33%), including two participants in Cohort 1, one participant each in Cohorts 2 and 3, and none in Cohort 4. The serious adverse events were lactic acidosis and nephrolithiasis (both in the same participant), metabolic disorder, cellulitis, and migraine (Table 3). The metabolic disorder event was described as transient glycemic instability in the setting of sinusitis. There was a single occurrence of each serious TEAE, and each recovered and/or resolved. The authors and study sponsor assessed all serious TEAEs as not related to study drug; all serious TEAEs were attributed to underlying GSDIa or pre‐existing medical conditions.

**TABLE 3:** Serious treatment‐emergent adverse events.

Preferred term	Cohort 1 (*N* = 3) *n* (%)	Cohort 2 (*N* = 3) *n* (%)	Cohort 3 (*N* = 3) *n* (%)	Cohort 4 (*N* = 3) *n* (%)	Total (*N* = 12) *n* (%)
Lactic acidosis	0 (0)	0 (0)	1 (33.3)	0 (0)	1 (8.3)
Metabolic disorder	1 (33.3)	0 (0)	0 (0)	0 (0)	1 (8.3)
Cellulitis	0 (0)	1 (33.3)	0 (0)	0 (0)	1 (8.3)
Migraine	1 (33.3)	0 (0)	0 (0)	0 (0)	1 (8.3)
Nephrolithiasis	0 (0)	0 (0)	1 (33.3)	0 (0)	1 (8.3)

Except for a Grade 3 serious TEAE of lactic acidosis in a participant in Cohort 3, all TEAEs were Grade 1 or 2 in severity. The TEAE of lactic acidosis occurred in the setting of metabolic instability, and intravenous dextrose was administered after the participant presented with a lactic acid concentration of 12.1 mmol/L. This event was deemed by the investigator to be attributable to GSDIa and not related to DTX401. No participant had metabolic decompensation due to corticosteroid therapy or adrenal crisis associated with corticosteroid withdrawal. No participant had a clinically significant change on non‐contrast liver magnetic resonance imaging or abdominal ultrasound during the study. In addition, there was no clinical change in renal function during the course of the study, with stable serum creatinine and 24‐h urinary protein excretion at 52 weeks post‐treatment compared with baseilne (Table S1).

Vector‐induced immunological reaction (e.g., increased ALT levels) in response to AAV administration is a known potential risk and adverse event of special interest for DTX401. All participants experienced mild or moderate TEAEs associated with liver enzyme elevations that were asymptomatic and resolved. No serious TEAE liver transaminase elevations were reported. In most participants, ALT reached peak values between Weeks 6 and 12. Study maximum ALT concentration reached 323 U/L on day 46 post‐DTX401 administration in participant #8. Generally, ALT values returned to individual baseline levels (or to within normal limits) during the 52‐week study. Eleven participants (all participants except one participant in Cohort 1) received prednisone at doses ranging from 40 to 60 mg/day for elevations of ALTs; mean prednisone administration duration for these 11 participants was 94 (range 42–199) days including the steroid wean; all discontinued immune suppression between 76 and 205 days after DTX401 dosing (Figure S1).

All participants were positive for binding and neutralizing antibodies to AAV8, but none were positive for anti‐G6Pase antibodies. Vector genome was detected in blood of all participants on Day 2 and gradually decreased over time, potentially representing a rapid distribution to tissues, followed by a redistribution over time. The excretion of viral vectors in saliva, urine, and stool was confirmed to be undetectable by Week 12 or earlier in all participants.

### 3.3 Efficacy Outcomes

DTX401 administration led to changes in dietary management, and CFCs provided new insights for the trial investigators that led to study protocol changes in how the CFCs were implemented. In one participant in Cohort 3, the CFC was stopped by the study investigator a few minutes after it started upon receipt of baseline results indicating clinically significant hyperlactatemia. Two other participants in Cohort 3 received approximately 5 g of cornstarch before each CFC, but their prefasting meal changed from carbohydrate content of 20 g and 34 g at baseline to personalized meals containing 20 g and 18 g of carbohydrates at Week 52, respectively. At baseline in Cohort 4, the glycemic threshold for stopping the CFC was 60 mg/dL, and all participants received 5 g of cornstarch; at the final CFC at Week 52, the glycemic threshold for stopping the CFC was 54 mg/dL (3.0 mmol/L), and participants received 40–50 g of cornstarch. In Cohort 4 participants, carbohydrate intake at the prefasting dinner was personalized and ranged from 18 to 20 g at baseline and from 5 to 23 g at Week 52.

**FIGURE 2:**
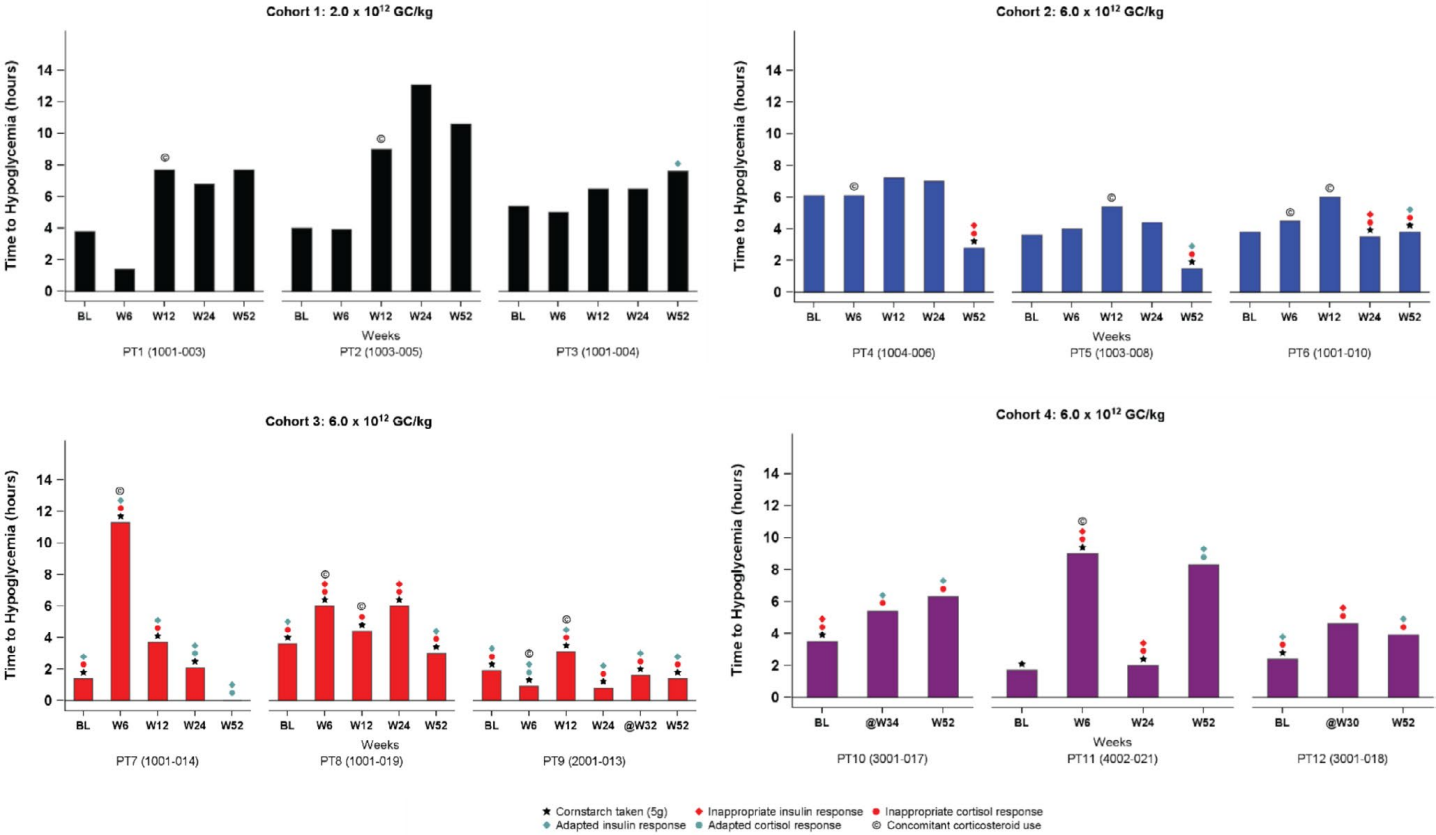
Individual participant time to hypoglycemia, insulin response, and cortisol response during the controlled fasting challenge at Baseline to Week 52. Insulin response was deemed inappropriate if ≥ 25 μIU/L at the start of the challenge or > 2 μIU/L at the end of the challenge. Cortisol response was deemed inappropriate if < 10 μg/dL at the end of the challenge. PT, participant.

Mean (standard deviation [SD]) time in hours to first hypoglycemic event during the CFC was 3.4 (1.4) at baseline, 5.9 (2.0) at Week 12 (mean [SD] increase, 2.2 [1.4]), 5.2 (3.5) at Week 24 (mean [SD] increase, 1.7 [2.8]), and 5.2 (3.1) at Week 52 (mean [SD] increase, 1.5 [3.3]). Individual participant time to hypoglycemia, insulin response, and cortisol response during the CFC are shown in Figure 2.

CFC results were also analyzed by calculating the time to hypoglycemia per unit of dietary carbohydrate (i.e., the sum of all carbohydrates and cornstarch) ingested before the CFC, expressed in minutes per gram of carbohydrate. This analysis corrected for the varying quantities of prechallenge carbohydrates administered under the different CFC protocols. Overall, the mean (SD) time in minutes/g of carbohydrate to hypoglycemia was 5.0 (1.6) at baseline, 7.3 (1.6) at Week 12 (mean [SD] increase, 2.8 [1.8]), 6.7 (3.4) at Week 24 (mean [SD] increase, 2.2 [3.2]), and 6.9 (2.7) at Week 52 (mean [SD] increase, 1.8 [3.1]). Mean (SD) percent increase in normalized time to hypoglycemia during the CFC from baseline to Week 52 was 46 (72).

**FIGURE 3:**
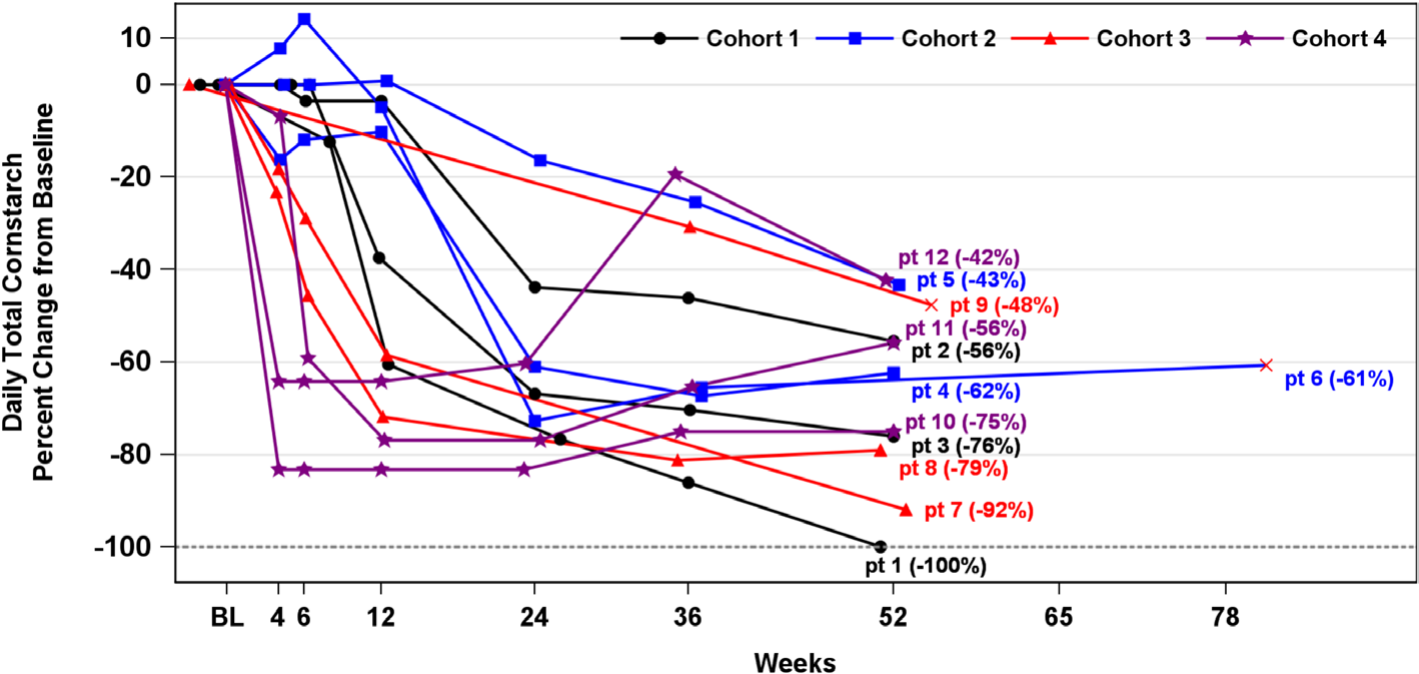
Individual participant daily total cornstarch intake reduction from baseline. Participant 6 (pt 6) Week 52 visit occurred at Week 81 due to a COVID‐19 study visit delay. Participant 9 (pt 9) Week 52 visit occurred at Week 55, which was outside the ±14‐day Week 52 visit window. pt., participant.

Daily cornstarch intake decreased from baseline in all participants. At baseline, cornstarch dose frequency for individual participants was between three and eight times daily, with total daily intake that ranged from 171 g (in Participant #2 who received overnight continuous gastric drip feeding and three daily 57 g cornstarch doses) to 405 g. At Week 52, the reduction in cornstarch intake for individual participants ranged from 42% to 100% in the 10 participants with available values at this timepoint. One participant did not take any cornstarch (100% reduction) at the completion of the study period. The mean (SD) cornstarch reduction was 68% (20%); *p* < 0.001 (Figure 3).

Similar to what was observed for daily actual cornstarch intake, the daily prescribed cornstarch also decreased from baseline for all participants. Six participants (50%) had their prescribed cornstarch intake frequency reduced by at least half from baseline, and five participants (42%) were prescribed two or fewer daily cornstarch doses. At Week 52, the reduction in prescribed cornstarch for individual participants ranged from 28% to 100%, with two participants not prescribed any cornstarch (100% reduction); mean (SD) prescribed cornstarch reduction was 70% (23%), and median cornstarch reduction was 75%. The decrease in daily prescribed cornstarch occurred more rapidly among participants who received Dose 2 (6.0 × 10^12^ GC/kg) in Cohort 3 with optimized prednisone and Cohort 4 with prophylactic prednisone compared with participants who received Dose 1 (2.0 × 10^12^ GC/kg) in Cohort 1. It should also be noted that participants in Cohorts 3 and 4 were those who used CGM, thereby allowing more rapid cornstarch dose adjustments. Changes in actual cornstarch intake were consistent with those for prescribed cornstarch intake, indicative of acceptable overall cornstarch compliance.

**FIGURE 4:**
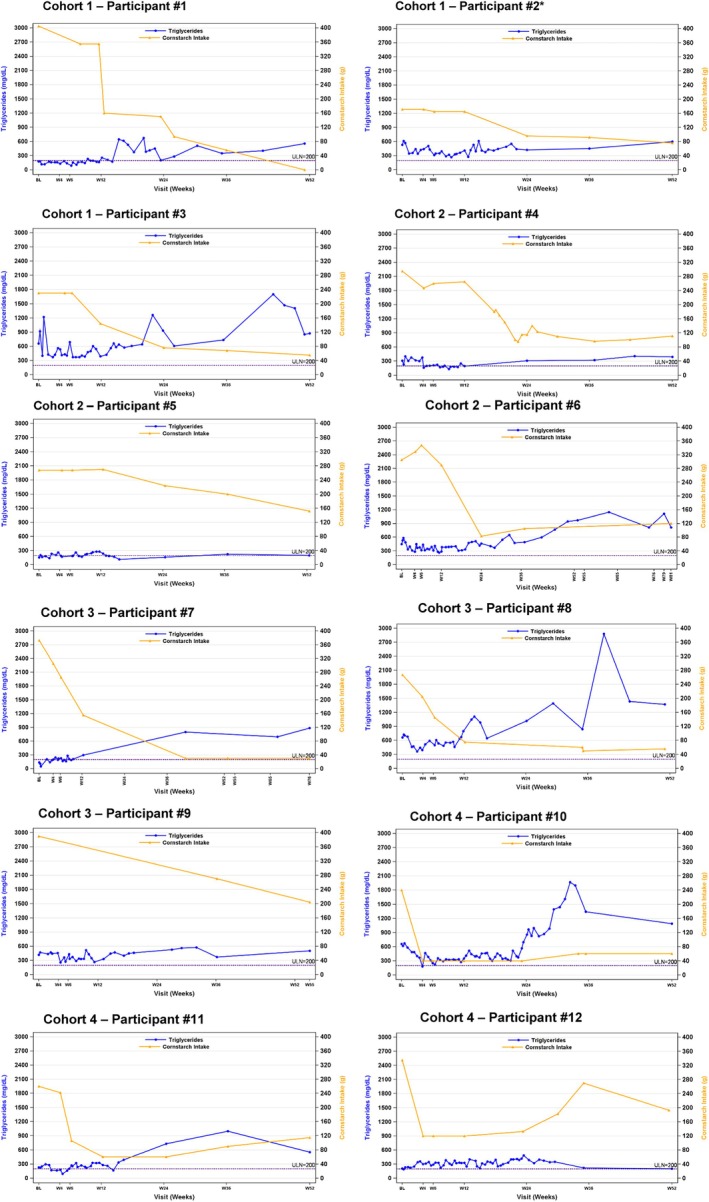
Individual participant triglycerides and actual daily cornstarch intake over time. *Participant #2 received overnight continuous gastric drip feedings.

Individual participant profiles for cornstarch intake and triglycerides are presented in Figure 4. Triglycerides initially decreased or were near baseline values until Week 12, then increased from baseline between Week 24 and Week 52. The mean (SD) change from baseline to Week 52 for triglycerides was 287 (254) mg/dL, with a median (range) change from baseline to Week 52 of 270 (−5, +749) mg/dL. The mean change from baseline to Week 52 for uric acid was 1.0 mg/dL (mean [SD] at baseline 5.91 [1.25] mg/dL versus 6.94 [2.29] mg/dL at Week 52), with a median (range) change from baseline to Week 52 of 0.65 (−1.2, +7.1) mg/dL.

**FIGURE 5:**
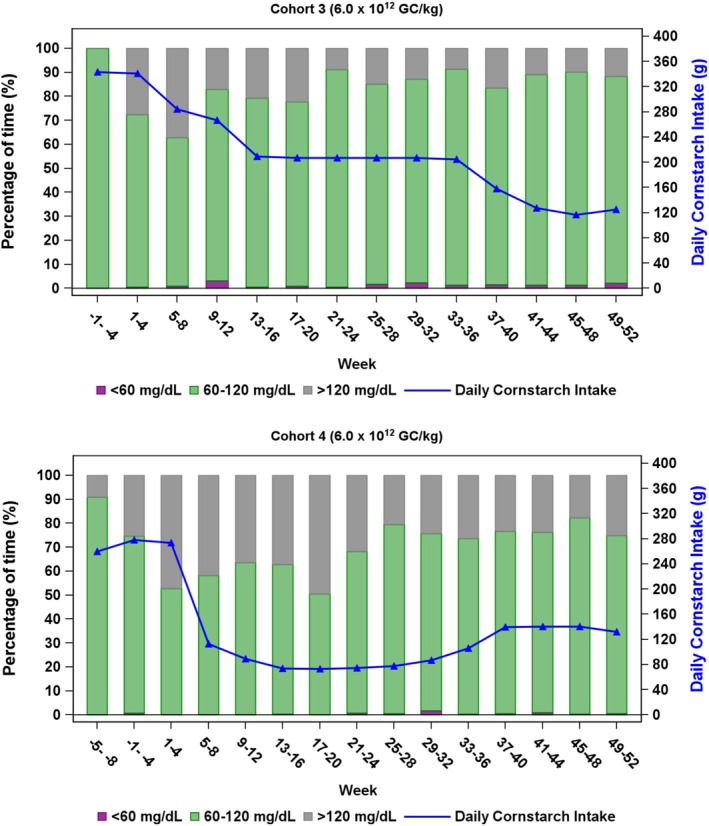
Average monthly (4 weeks) percentage of time spent in glucose range < 60, 60–120, and > 120 mg/dL with average daily actual cornstarch intake by cohort.

Results for CGM were available starting at approximately the baseline visit for participants in Cohorts 3 and 4 (Figure 5). In Cohort 3, average monthly time in euglycemia (protocol‐defined as 60 to 120 mg/dL) increased from 72% at baseline (Weeks 1 to 4) to 86% at the end of study (Weeks 49–52), despite a 60% reduction in average daily cornstarch intake (from 288 to 115 g). In Cohort 4, average monthly time in euglycemia remained stable from baseline (74%) to the end of study (75%), despite a 52% reduction in average daily cornstarch intake (from 273 to 132 g). Hypoglycemic episodes at night (between 12:00 am and 6:00 am) were rare. In addition, seven participants from Cohorts 1, 2, and 3 provided results for morning glucose levels during at least 1 week of the study; in these participants, weekly morning mean glucose levels were mostly normoglycemic (60 to 120 mg/dL), very few (~7%) were > 120 mg/dL, and none were < 60 mg/dL.

### 3.4 Patient‐Reported Quality of Life

Of the nine participants in Cohorts 2, 3, and 4 who completed the PGIC, five (56%) reported GSDIa was “much improved”, one (11%) reported GSDIa was “moderately improved”, two (22%) reported GSDIa was “minimally improved”, and one (11%) reported “no change” from baseline at Week 52. No participant reported worsening of GSDIa assessed using the PGIC despite reduced frequency and intake of daily cornstarch.

The six participants in Cohorts 3 and 4 who completed the PGIS reported generally similar results at baseline and Week 52 for episodes of low blood sugar, general health, the severity of GSDIa symptoms, and the impact of GSDIa on their life over the prior 7 days. Lack of baseline data for participants enrolled in Cohorts 1 and 2 limited interpretation of these results.

## 4 Discussion

In this gene therapy trial in patients with GSDIa, first‐in‐human administration of DTX401 showed an acceptable safety profile across all dose cohorts, with no DLTs, TEAEs leading to study discontinuation, TEAEs leading to death, or serious treatment‐related TEAEs. While there was some metabolic instability following administration of the vector, severe hypoglycemia was avoided, and there was no progression of long‐term complications in the 52‐week follow‐up period. All participants demonstrated clinically meaningful reductions in cornstarch intake, and most described improvements in patient‐reported quality of life. In people with GSDIa, conventional diets cause glycemic instability, and the inability to mobilize glucose from stored glycogen makes people with GSDIa dependent on a continuous supply of exogenous carbohydrates. To avoid life‐threatening hypoglycemia, large quantities of cornstarch are administered. After DTX401 administration, participants in this trial successfully weaned cornstarch, indicative of increased endogenous glucose production. This is critical since acute hypoglycemia is a primary risk for people with GSDIa, and the ability to mobilize hepatic glycogen would minimize the risk of severe hypoglycemia, seizures, and death.

It is important to recognize that this trial had challenges that distinguish it from other liver‐directed gene therapy development programs. First, in contrast with other conditions such as hemophilia, disease severity and therapeutic monitoring of patients with GSDIa cannot rely on measuring circulating protein as a surrogate for transgene activity [21]. Second, GSDIa efficacy outcome measures are either counterintuitive and potentially dangerous, or lack recognition as standard of care from agreed‐upon, universal treatment guidelines. Third, immunosuppression with prednisone was administered to preserve DTX401‐expressing hepatocytes, but corticosteroids in GSDIa impact glycemic and metabolic control, causing hyperlactatemia and hyperlipidemia. Fourth, delivery of the gene to the liver allowed stored glycogen to be released, and this resulted in post‐treatment hyperglycemia, relative hyperinsulinism, and rebound hypoglycemia which was exacerbated by the steroid administration. Finally, GSDIa is a rare disorder with heterogeneity caused by both the *G6PC1* genotype and additional genetic and/or environmental modifying factors [22].

In preclinical models of GSDIa, 3%–10% of normal G6Pase activity was required to normalize glucose concentrations with fasting, but 7% to 20% of normal activity was required to normalize lactate and lipid concentrations [23, 24]. G6Pase enzyme activity levels between these two thresholds may explain the elevated triglycerides documented in some of the participants at week 52 and may be an important consideration when modifying cornstarch therapy after DTX401 administration. While all participants successfully weaned cornstarch support over the course of the study, variability in both the tolerated treatment wean and the duration of fasting occurred. Some variability in response to DTX401 may be due to differences in *G6PC1* genotypes. The p.Arg83Cys pathogenic variant has been shown to be metabolically more severe than other GSDIa pathogenic variants with partial enzyme activity [25, 26]. Patients with variants associated with nonfunctional G6Pase activity in vitro, including those with p.Arg83Cys (homozygous participants in Cohorts 3 and 4), may require additional management strategies since enzymatic activity above the aforementioned thesholds may not be achieved [25, 26].

Comparing gene therapies and the optimal duration of corticosteroid therapies between clinical trials must be done cautiously, given differences between disease states and transgene doses. For example, in a gene therapy trial of an AAV8 vector in 10 patients with hemophilia B, participants received transgene doses ranging from 2.0 × 10^11^ to 2.0 × 10^12^ vector genomes (VG) per kg of body weight; four of six participants in the high dose group had increased ALT levels and received a tapering dose of prednisolone, and elevated ALTs resolved over a range of 2–35 days (median 5 days) [27, 28]. In another gene therapy study of an AAV5 vector in 54 patients with hemophilia B, participants received a transgene dose of 2.0 × 10^13^ GC/kg; glucocorticoid therapy was necessary in nine participants with elevated ALT levels and was administered over a range of 51–130 days (mean 79.8 days) [21]. Across five gene therapy trials of an AAV9 vector in 100 patients with spinal muscular atrophy, all but three participants (who received a lower dose) received a transgene dose of 1.1 × 10^14^ VG/kg; prednisolone was administered to all participants in these trials beginning 1 day before AAV9 administration and was continued for at least 30 days [29]. Following an acute liver failure event, the prednisolone regimen was modified to start 3 days before AAV9 administration and continued for 30 days, followed by a steroid taper. In these trials, prednisolone was administered over a range of 33–229 days (mean 83 days) [29].

In our trial, due to the known challenges of administering steroids in GSDIa, we used different approaches for corticosteroid therapy. Attempts to minimize required steroids led to multiple protocol amendments and study modifications. Compared with other trials and based on the direct effect of prednisone on the regulation of the *G6PC1* transgene, corticosteroid therapy may have impacted glucose levels in the first 8‐12 weeks after DTX401 administration. After Week 24, when the effect of corticosteroids on glucose homeostasis was minimal, we observed a sustained reduction in cornstarch doses that was then mantained throughout the rest of the 52‐week study. The large reduction in cornstarch requirements was accompanied by autonomous stable or improved glycemic control, which could only occur if glucose mobilization was improved as a result of *G6PC1* transgene activity.

The DTX401 development program also differs from other gene therapies because of the need for frequent adjustments in medical management during the first 3–6 months after DTX401 administration. The combined effects of DTX401 and corticosteroid administration require close monitoring of glucose homeostasis and changes in dietary management to minimize both hyperglycemia and rebound hypoglycemia. Quantitating the immune response to the vector can also be challenging in patients with GSDIa due to difficulties with interpreting liver function parameters: ALT can be increased by a combination of impaired metabolic control (due to diet and corticosteroids) and immune response to the AAV8 vector. In addition, glycemic control assessment using increased hepatic G6Pase activity can only be indirectly assessed in the absence of invasive liver biopsies and is estimated primarily by changes in cornstarch requirements. Furthermore, observational studies in small animal models and in patients with GSDIa have demonstrated that deficient hepatic G6Pase activity does not necessarily reflect that endogenous hepatic glucose production is zero [30, 31, 32, 33, 34, 35, 36].

CGM has been shown to be an effective tool for the measurement of blood glucose in glycogen storage disease patients [18, 19, 37, 38]. In our study, CGM was not initially included since it was not part of standard clinical practice, but CGM was implemented in Cohorts 3 and 4 as it became standard of care for the field. CGM use showed generally stable glucose levels throughout the day, although hyperglycemia could occur when cornstarch was weaned too slowly, presumably due to the combination of starch and endogenous glucose release from the liver. Nocturnal hypoglycemia was rare despite reductions in cornstarch intake. There was a discordance between the time to hypoglycemia during the inpatient CFC and the observation that participants could sleep through the night without experiencing hypoglycemia per CGM data when they were at home; this could have been due to inaccuracies that may be inherent to CGM devices or deviations from normal diet and activity associated with inpatient care. It is also unclear whether time to hypoglycemia in the hospital setting predicts the occurrence of hypoglycemia at home.

During the CFC, inappropriate insulin and cortisol counterregulatory responses were documented with relative hyperinsulinism in the setting of physiologic hyperglycemia and abonormally low cortisol concentrations in the setting of hypoglycemia. These findings require further investigation and may have also confounded the interpretation of CFC results. Investigators noted that participants were motivated to reduce cornstarch intake, highlighting the burden of nutritional management; in the future, patients with GSDIa treated with DTX401 should be educated to remain vigilant with nutritional management to minimize disease complications.

There were many lessons learned in the course of the study which resulted in various protocol amendments. First, the chronic intake of carbohydrate required for treatment of GSDIa likely contributes to abnormal insulin secretion. Immunosuppression was initially used only in the setting of hepatic transaminase elevation, but it was later deemed necessary before the presence of inflammation; this resulted in modification of the protocol and the creation of Cohorts 3 and 4. Second, signs and symptoms of hypoglycemia associated with an adrenergic reaction and cerebral glucopenia and most physiological hormonal and metabolic responses to hypoglycemia might not occur in participants with GSDIa until they reach blood glucose levels < 60 mg/dL (< 3.3 mmol/L) [4, 39]. This resulted in lowering the glucose stopping threshold during the CFCs in Cohort 4 to < 54 mg/dL (< 3.0 mmol/L). Finally, hyperlipidemia related to metabolic instability following therapy warrants more investigation. While high triglycerides are well known to occur in GSDIa patients in the setting of hypoglycemia and glycogenolysis, one of the lessons from this study is that over‐treatment and associated metabolic instability is also associated with hyperlipidemia.

Limitations of our study include that GSDIa is a rare genetic disorder with a low incidence frequency in the general population that, by necessity, results in a small sample size and a single‐arm trial when exploring a therapy in a Phase 1/2 study. Limitations also include the modifications to the conduct of CFCs that included changes in pre‐fasting cornstarch doses and dietary carbohydrate intake. These changes complicated the interpretation of time to hypoglycemia as an efficacy outcome parameter; however, despite changes to CFC conduct, we observed increases in time to hypoglycemia in some participants. Normalization of time to hypoglycemia helped reduce this confounding; however, not all carbohydrates are digested and metabolized at the same rate. It should be noted that we learned from this rare disorder while studying it, which necessarily led to these protocol amendments.

In conclusion, gene therapy with DTX401 in participants with GSDIa had an acceptable safety profile across all dose cohorts, with no DLTs, TEAEs leading to study discontinuation, TEAEs leading to death, or serious treatment‐related TEAEs. Prophylactic and reactive use of oral corticosteroids mitigated the known potential risk of vector‐induced inflammatory response. DTX401 was associated with maintained or improved glycemic stability that was sustained through Week 52, despite reductions in both the frequency and intake of daily cornstarch. Time to hypoglycemia in the CFC increased after DTX401 administration. Most participants reported improvements in health‐related quality of life, and no participant reported worsening of GSDIa despite reduced frequency and intake of daily cornstarch. The DTX401 dose of 6.0 × 10^12^ GC/kg by qPCR (equivalent to 1.0 × 10^13^ GC/kg by droplet digital polymerase chain reaction) was selected for further development in a phase 3 trial (NCT05139316).

## Author Contributions

All authors had full access to all the data in the study. All authors collected and interpreted the data. Deepali Mitragotri analyzed the data. David A. Weinstein and Deepali Mitragotri directly accessed and verified the underlying data reported in the manuscript. David A. Weinstein, Terry G. Derks, David F. Rodriguez‐Buritica, Ayesha Ahmad, María‐Luz Couce, John J. Mitchell, Rebecca Riba‐Wolman, Deepali Mitragotri, and Eric Crombez wrote the original draft of the manuscript. All authors reviewed and edited subsequent drafts and accept responsibility to submit the manuscript for publication.

## Ethics Statement

The protocol was approved by the institutional review board at each participating center. All procedures followed were in accordance with the ethical standards of the responsible committee on human experimentation (institutional and national) and with the Helsinki Declaration of 1975, as revised in 2000. Informed consent was obtained from all patients for being included in the study.

## Conflicts of Interest

For all authors, Ultragenyx provided the investigational agent, grant support to their institutions for the conduct of this clinical trial, and medical writing support in the preparation of this manuscript. David A. Weinstein served on the scientific advisory board for Dimension Therapeutics prior to the acquisition of Dimension by Ultragenyx and is an unpaid member of the board of directors of the Association for Glycogen Storage Disease. Terry G. Derks reported financial research support for investigator‐initiated research and sponsor‐initiated research from Ultragenyx, consulting fees from Danone, Ultragenyx, ModernaTX Inc., and Beam Therapeutics, honoraria for lectures or presentations on behalf of MEDTalks, Prelum, Danone, and participation on a data safety monitoring board/advisory board for ModernaTX Inc. David F. Rodriguez‐Buritica received consulting fees from Ultragenyx, Moderna, and Beam Therapeutics. John J. Mitchell received consulting fees from Biomarin, Takeda, and Sanofi‐Genzyme, payment or honoraria from Biomarin and Takeda, and participated on a data monitoring committee for Moderna. Rebecca Riba‐Wolman received consulting fees from Beam Therapeutics and payment or honoraria from Ultragenyx. Malaya Mount received consulting fees from ModernaTX Inc, Beam Therapeutics, and Danone, as well as travel support for attending meetings from ModernaTX Inc and Danone. Heather Saavedra received consulting fees from Danone (paid to institution), ModernaTX Inc., Beam Therapeutics, Sanofi, Vitaflo International, and payment or honoraria and support for attending meetings from Ultragenyx. Deepali Mitragotri is an employee and shareholder of Ultragenyx. Eric Crombez is an employee and shareholder of Ultragenyx. Ayesha Ahmad, María‐Luz Couce, Julieta Bonvin Sallago, Katalin M. Ross, Melanie M. van der Klauw, Foekje de Boer, Caroline van der Schaaf, Miguel Martínez‐Olmos, Elvis Atanga, and Asad Hosseini authors declare no conflicts of interest.

## Supporting information


**Data S1.** Supporting Information.

## Data Availability

Deidentified participant data is available from the sponsor (publications@ultragenyx.com) upon reasonable request with a signed data access agreement. The redacted clinical study protocol and statistical analysis plan are available at ClinicalTrials.gov Identifier: NCT03517085.
