# Supplementary material for: Safety and Efficacy of DTX401, an AAV8‐Mediated Liver‐Directed Gene Therapy, in Adults With Glycogen Storage Disease Type I a (GSDIa)
Source: J Inherit Metab Dis. 2025 Mar 10;48(2):e70014. doi: 10.1002/jimd.70014 (PMC11893205; doi:10.1002/jimd.70014)

**Supplementary Appendix**

**TRIAL ENDPOINTS**

As the trial progressed, additional learnings led to CFC methodology modifications. In Cohort 1, all participants received 35 g of cornstarch at each challenge, with no change in total carbohydrate intake offered in the prefasting challenge dinner meal from what was consumed at the baseline CFC; the threshold for hypoglycemia at each challenge was 60 mg/dL (3.3 mmol/L). Sample collection to determine insulin and cortisol levels during the CFC was added to the trial protocol after Participants 1 and 2 completed the study, which corresponded to Week 52 for Participant 3. After review of Week 12 data from participants enrolled in Cohort 2, to normalize baseline glucose levels and minimize potential rebound hypoglycemia, the recommended target carbohydrate range for the prefasting challenge dinner meal was decreased to a target range of 15 g to 20 g from a range of 20 g to 30 g, and the prefasting challenge cornstarch dose was decreased from 35 g to 5 g. After changing the prefasting cornstarch dose to 5 g, further analysis and review of fasting challenge data showed shorter fasting times in Cohorts 2 and 3; this resulted in an additional change in requirements where the dinner meal was personalized for each participant. This personalized meal included a target carbohydrate range, with an overall composition in protein, fats, and dietary fiber as close as possible to the most current dinner prescription, but not higher than the carbohydrate content of the dinner consumed at the baseline fasting challenge for each participant. The threshold for stopping the CFC was also lowered to 54 mg/dL (3.0 mmol/L) or to when the participant presented with signs and/or symptoms of hypoglycemia. Lowering the stopping threshold was based on the signs and symptoms of hypoglycemia associated with an adrenergic reaction and cerebral glucopenia, while increasing metabolic pathways such as gluconeogenesis, fatty acid oxidation, and ketogenesis, since most physiological hormonal and metabolic responses to hypoglycemia might not occur in patients with GSDIa until they reach these blood glucose levels.

Table S1. Serum creatinine and 24-hour urinary protein excretion at Baseline and Week 52

|  | Baseline  N=12 | | Week 52  N=12 | |
| --- | --- | --- | --- | --- |
| Serum creatinine (mg/dL), mean (SD) | 0.72 (0.43) | 0.68 (0.47) | |  |
| 24-hour urinary protein (mg/dL), mean (SD) | 17.7 (27.6) | 19.6 (22.2) | |  |

**Figure S1. Individual participant alanine aminotransferase and prednisone intake over time**

**Cohort 1 – Participant #1**


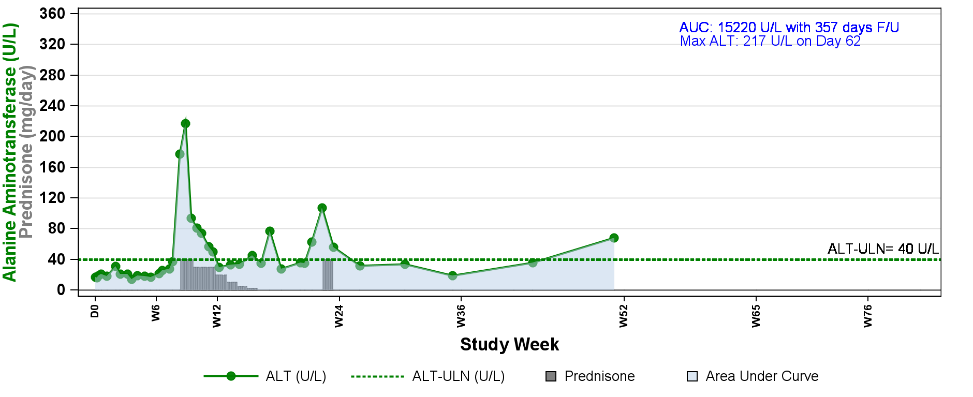


**Cohort 1 – Participant #2**


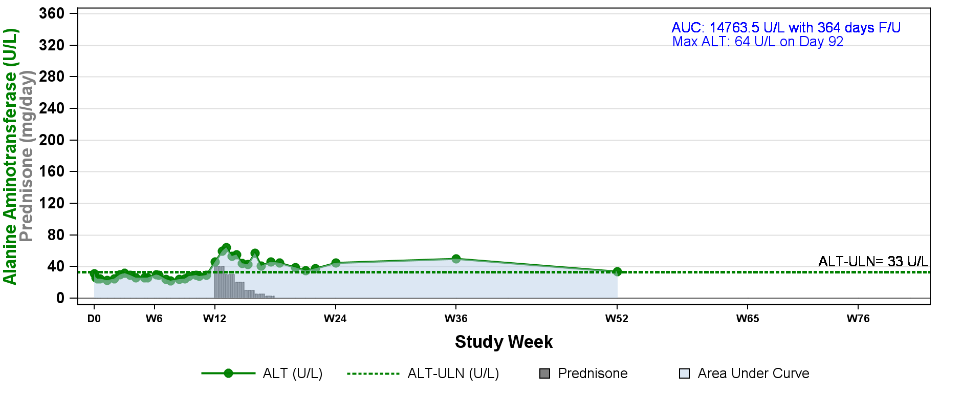


**Cohort 1 – Participant #3**


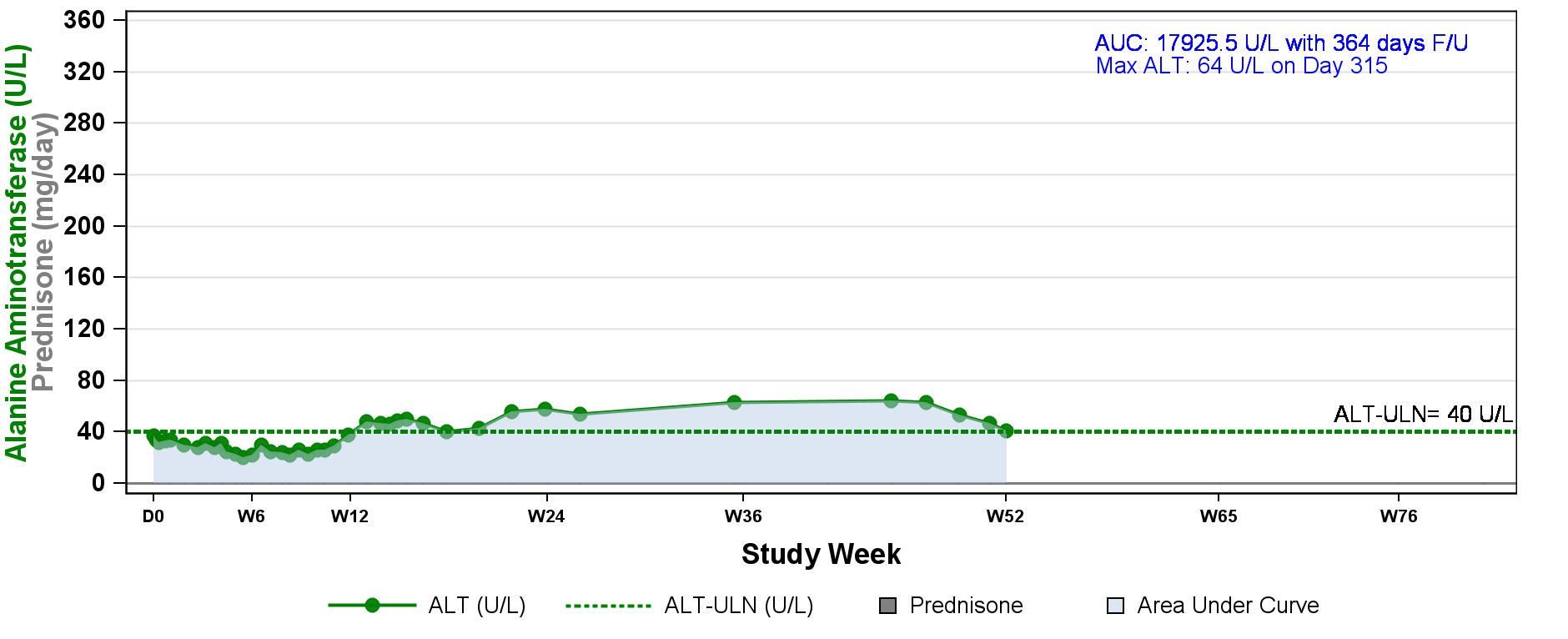


**Cohort 2 – Participant #4**


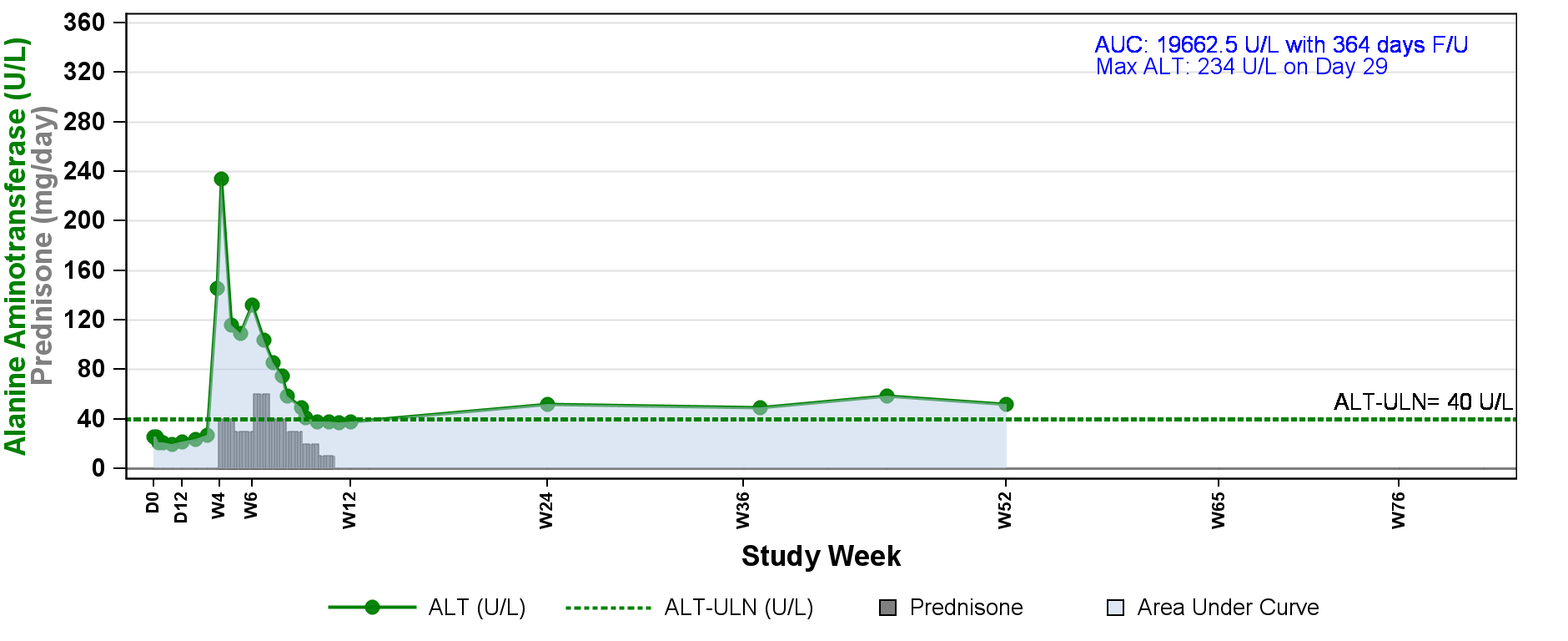


**Cohort 2 – Participant #5**


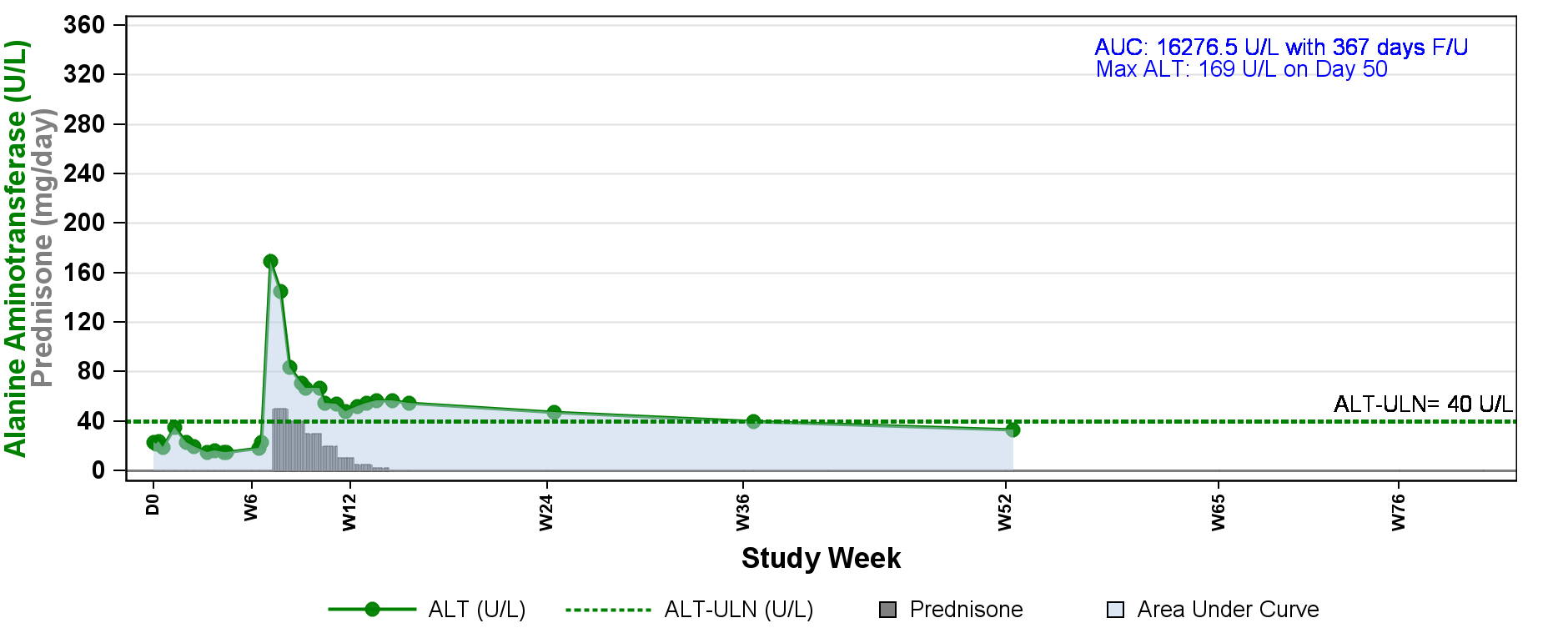


**Cohort 2 – Participant #6**


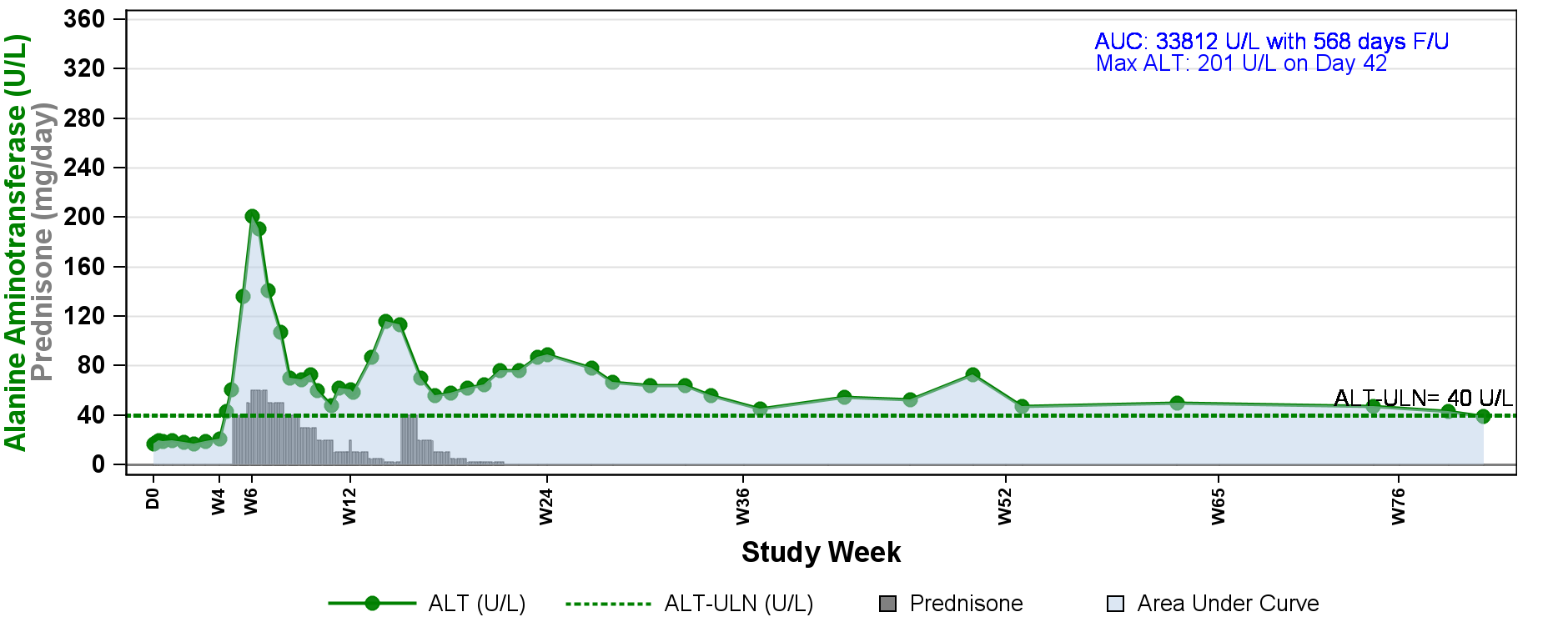


**Cohort 3 – Participant #7**


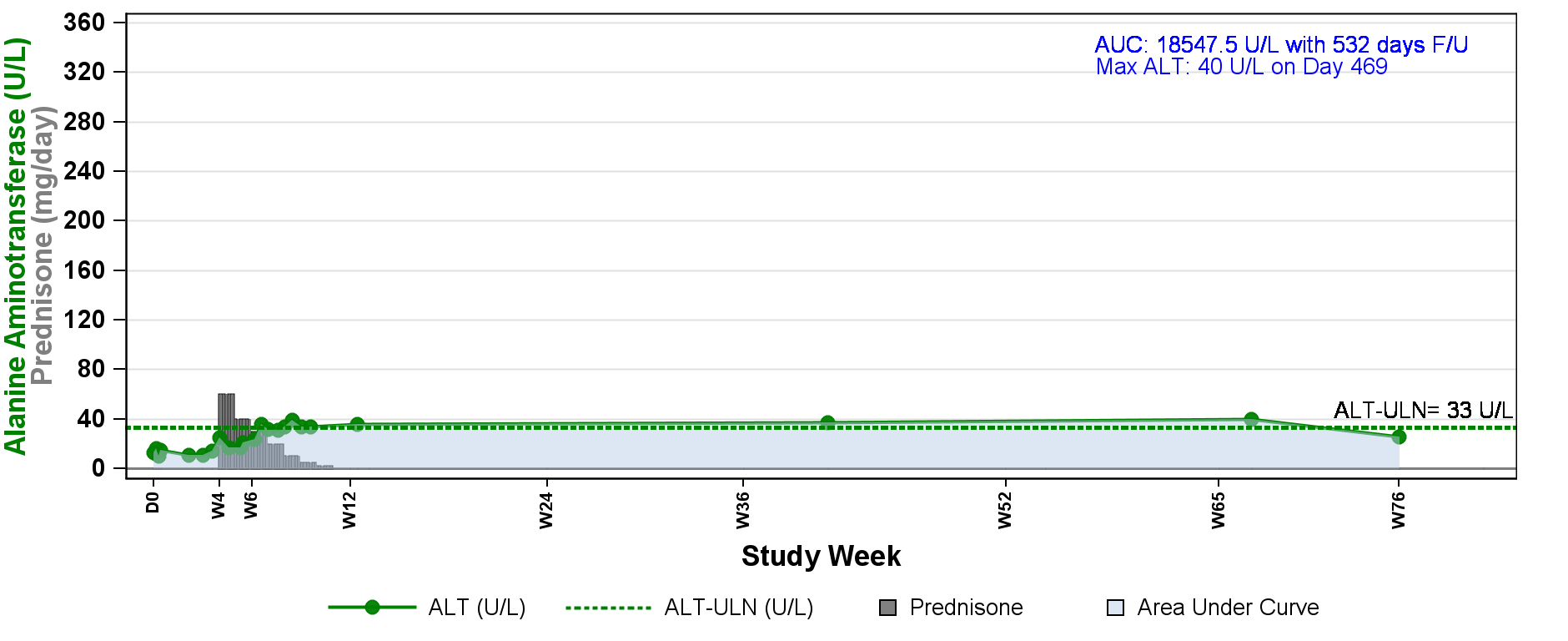


**Cohort 3 – Participant #8**


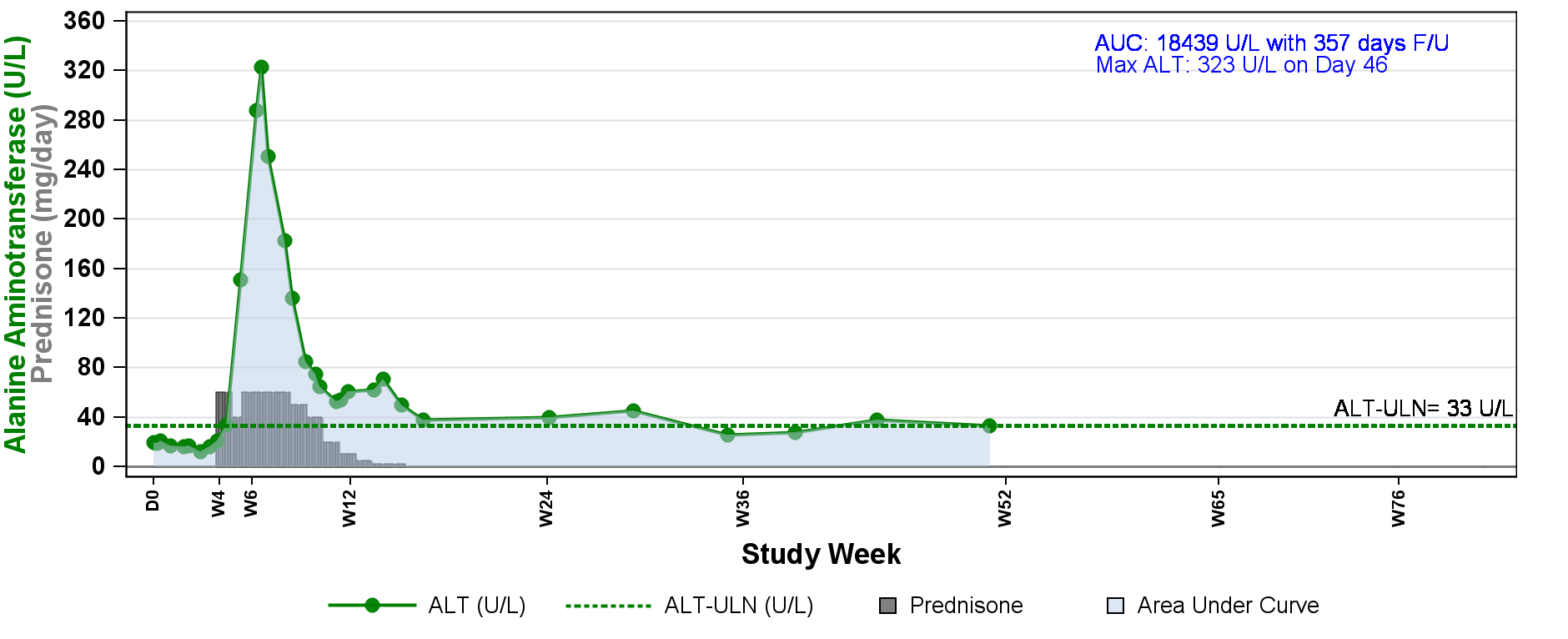


**Cohort 3 – Participant #9**


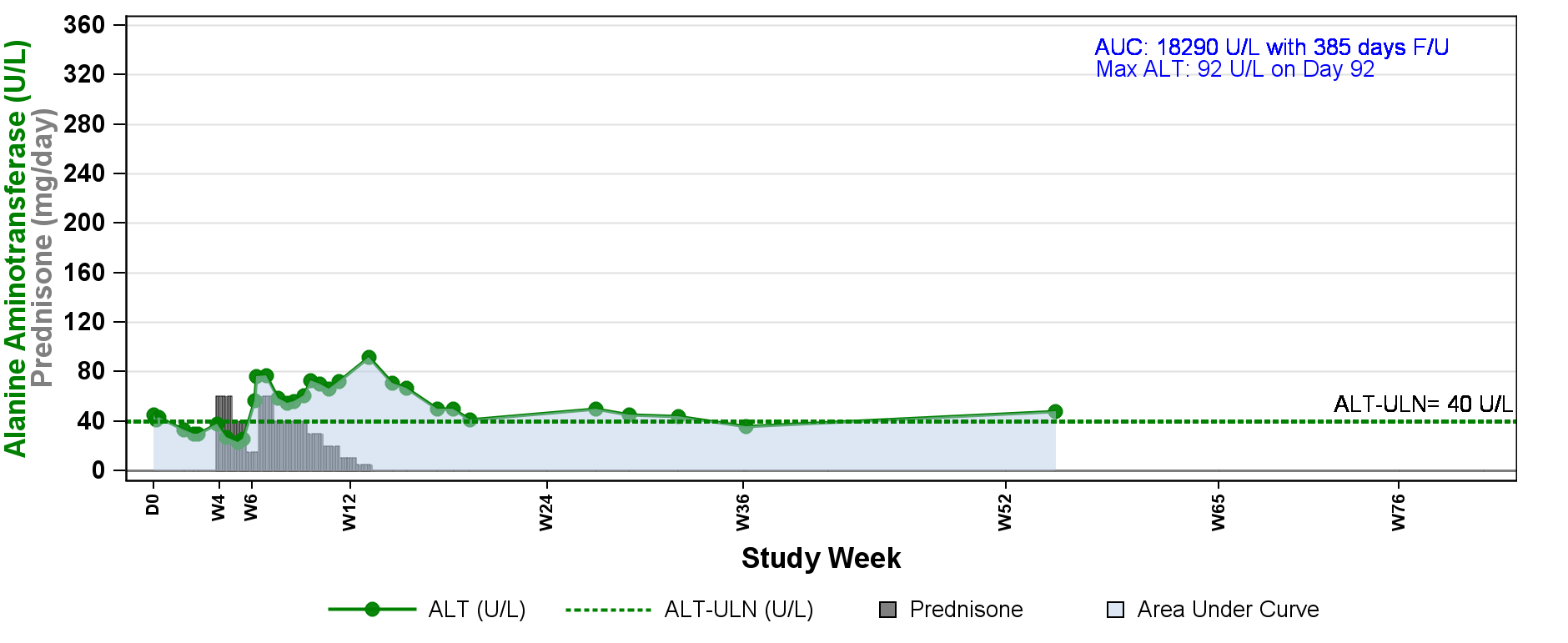


**Cohort 4 – Participant #10**


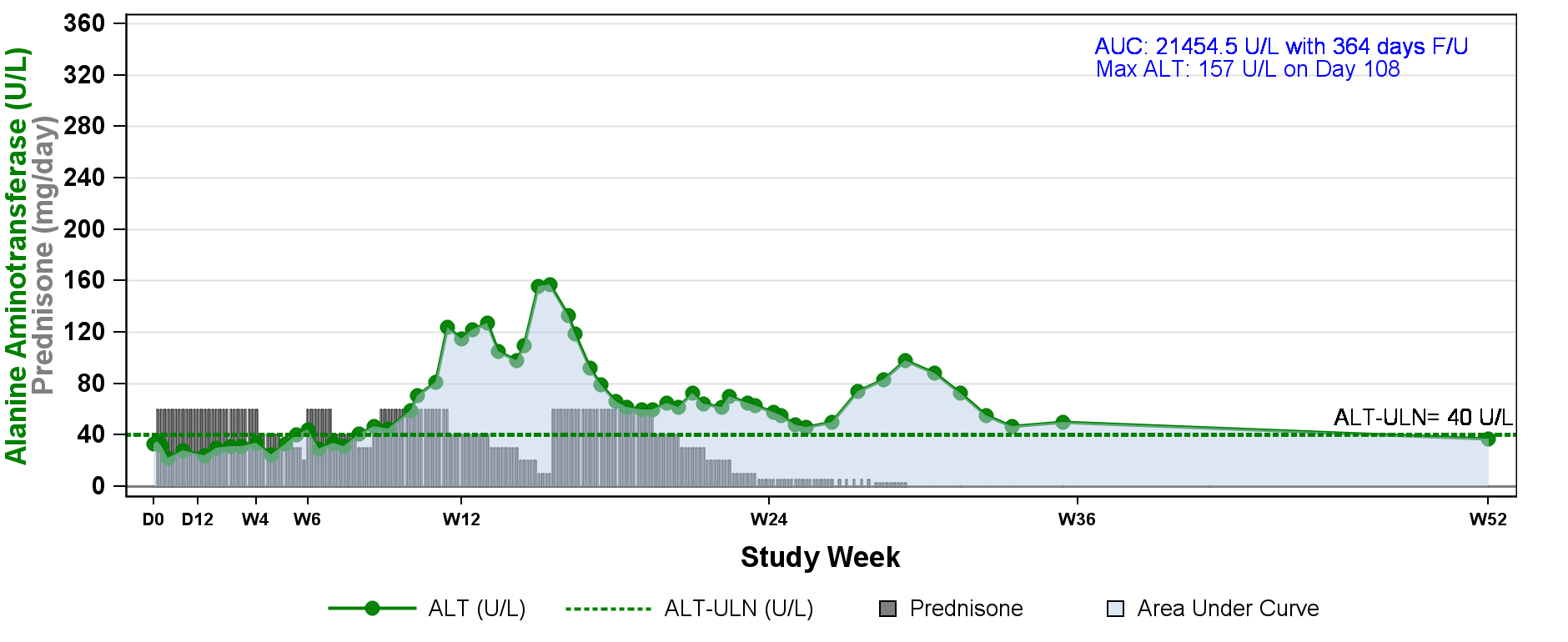


**Cohort 4 – Participant #11**


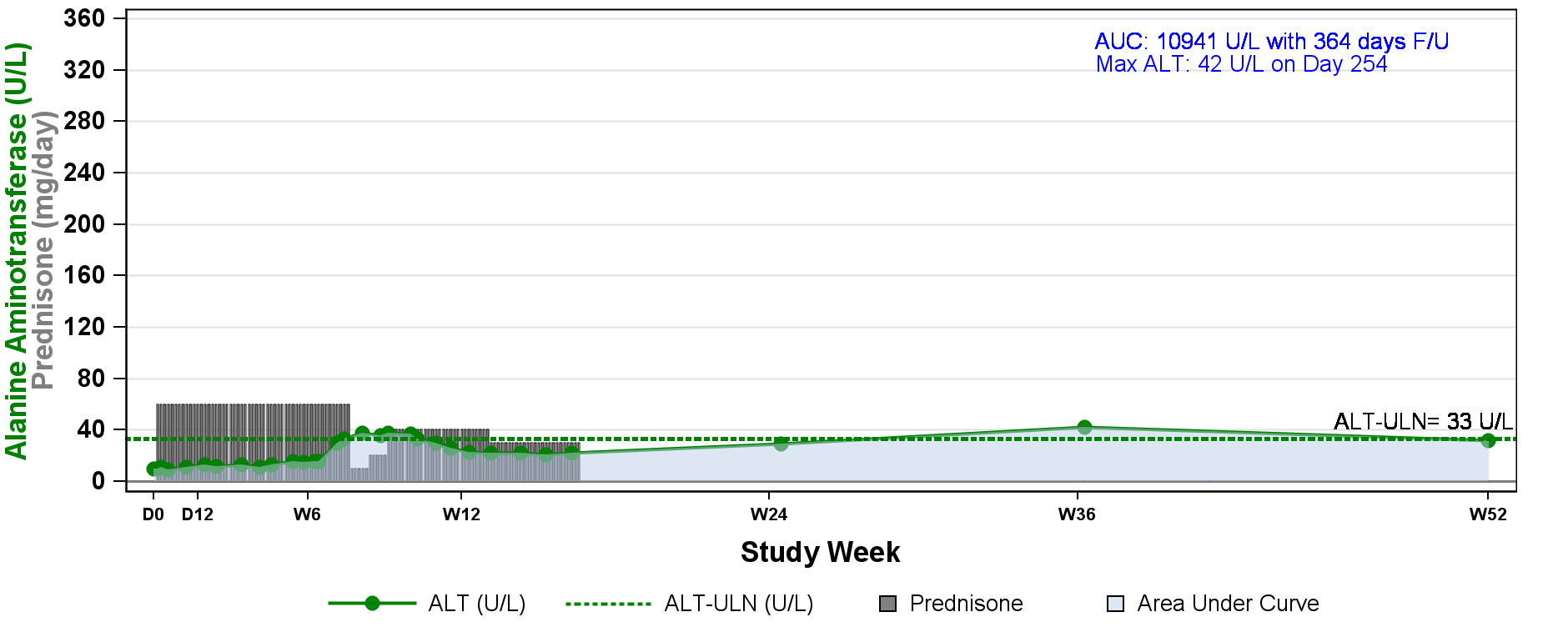


**Cohort 4 – Participant #12**


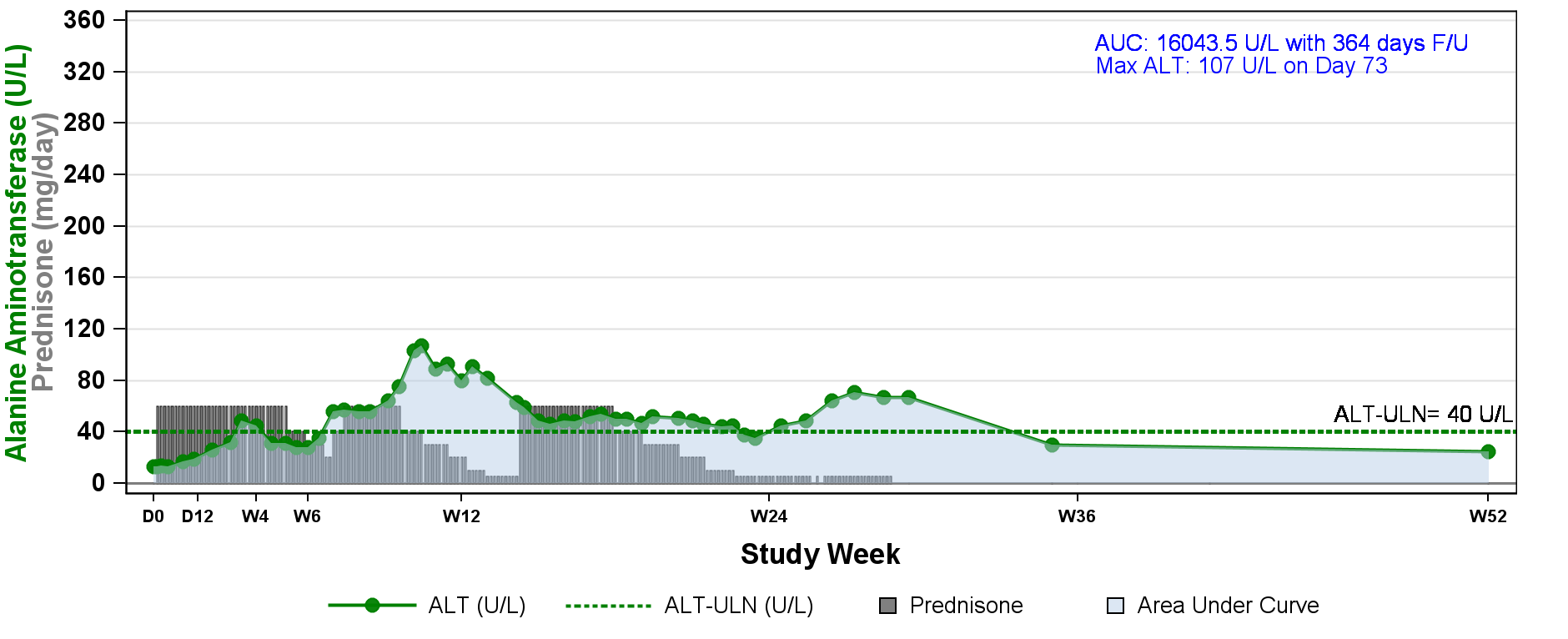

Supplement: Supplementary file 1 — Data S1. Supporting Information. [file JIMD-48-0-s001.docx]
